# Synergistic induction of ICAM-1 expression by cisplatin and 5-fluorouracil in a cancer cell line via a NF-**κ**B independent pathway

**DOI:** 10.1038/sj.bjc.6690449

**Published:** 1999-06

**Authors:** K Takizawa, R Kamijo, D Ito, M Hatori, K Sumitani, M Nagumo

**Affiliations:** Second Department of Oral and Maxillofacial Surgery, School of Dentistry, Showa University, 2-1-1 Kitasenzoku, Ota-ku, Tokyo, 145-8515, Japan

**Keywords:** CDDP, 5-FU, ICAM-1, NF-κB, protein tyrosine kinase

## Abstract

Cisplatin (CDDP) and 5-fluorouracil (5-FU) are common anti-tumour agents, and the anti-tumour effect of CDDP and 5-FU are synergistically enhanced by combined treatment. To clarify the mechanisms of this synergism, we examined the effect of CDDP and 5-FU on the expression of cell adhesion molecules involved in recognition of cancer cells by T lymphocytes. When NA cells, a squamous cell carcinoma cell line, were exposed to CDDP and 5-FU for 18 h, the expression of intercellular adhesion molecule-1 (ICAM-1) was synergistically induced, whereas CDDP or 5-FU alone did not induce the expression of ICAM-1, as determined by flow cytometry. Expression of ICAM-2 and ICAM-3, which are recognized by the same counter receptor on T-cells, were not up-regulated by CDDP and 5-FU. RT-PCR analysis showed that the induction of ICAM-1 on NA cells might be due to transcriptional induction of ICAM-1 mRNA. Treatment with genistein, a protein tyrosine kinase (PTK) inhibitor, inhibited the induction of ICAM-1 on NA cells by CDDP and 5-FU, whereas staurosporin, a protein kinase C inhibitor, did not. Although CDDP and 5-FU induced binding at the nuclear factor kappa B (NF-κB) site in the ICAM-1 promoter, pretreatment with genistein did not prevent CDDP and 5-FU-induced binding at the NF-κB site. Moreover, a NF-κB nuclear translocation inhibitor did not inhibit the induction of ICAM-1 expression by treatment with CDDP and 5-FU. The synergistic effect of CDDP and 5-FU was not specific to NA cells, since ICAM-1 was synergistically induced by CDDP and 5-FU on HSC-4 cells, a squamous cell carcinoma cell line. These findings indicate that treatment with CDDP and 5-FU induces ICAM-1 expression by a NF-κB independent regulatory mechanism involving PTK. © 1999 Cancer Research Campaign


*British Journal of Cancer* (1999) **80**(7), 954–963
© 1999 Cancer Research Campaign
Article no. bjoc.1998.0449

### Synergistic induction of ICAM-1 expression by cisplatin and 5-fluorouracil in a cancer cell line via a NF-κB independent pathway

**K Takizawa, R Kamijo, D Ito, M Hatori, K Sumitani and M Nagumo**

Second Department of Oral and Maxillofacial Surgery, School of Dentistry, Showa University, 2-1-1 Kitasenzoku, Ota-ku, Tokyo 145-8515, Japan

**Summary** Cisplatin (CDDP) and 5-fluorouracil (5-FU) are common anti-tumour agents, and the anti-tumour effect of CDDP and 5-FU are synergistically enhanced by combined treatment. To clarify the mechanisms of this synergism, we examined the effect of CDDP and 5-FU on the expression of cell adhesion molecules involved in recognition of cancer cells by T lymphocytes. When NA cells, a squamous cell carcinoma cell line, were exposed to CDDP and 5-FU for 18 h, the expression of intercellular adhesion molecule-1 (ICAM-1) was synergistically induced, whereas CDDP or 5-FU alone did not induce the expression of ICAM-1, as determined by flow cytometry. Expression of ICAM-2 and ICAM-3, which are recognized by the same counter receptor on T-cells, were not up-regulated by CDDP and 5-FU. RT-PCR analysis showed that the induction of ICAM-1 on NA cells might be due to transcriptional induction of ICAM-1 mRNA. Treatment with genistein, a protein tyrosine kinase (PTK) inhibitor, inhibited the induction of ICAM-1 on NA cells by CDDP and 5-FU, whereas staurosporin, a protein kinase C inhibitor, did not. Although CDDP and 5-FU induced binding at the nuclear factor kappa B (NF-κB) site in the ICAM-1 promoter, pretreatment with genistein did not prevent CDDP and 5-FU-induced binding at the NF-κB site. Moreover, a NF-κB nuclear translocation inhibitor did not inhibit the induction of ICAM-1 expression by treatment with CDDP and 5-FU. The synergistic effect of CDDP and 5-FU was not specific to NA cells, since ICAM-1 was synergistically induced by CDDP and 5-FU on HSC-4 cells, a squamous cell carcinoma cell line. These findings indicate that treatment with CDDP and 5-FU induces ICAM-1 expression by a NF-κB independent regulatory mechanism involving PTK.

**Keywords:** CDDP; 5-FU; ICAM-1; NF-κB; protein tyrosine kinase

Chemotherapy is widely used as adjuvant therapy for the treatment of squamous cell carcinoma (SCC). 5-Fluorouracil (5-FU) is increasingly used for the treatment of SCC, though it sometimes has a low efficacy when administered alone. However, various drug combinations, especially 5-FU with cisplatin (CDDP), have been effective in cancer treatment (Kish et al, 1982; Amrein and Weitzman, 1985; Schilsky et al, 1989). Preclinical studies have revealed that these drugs administered in combination demonstrate synergistic anti-tumour activity (Scanlon et al, 1986; Pratesi et al, 1988; Kuroki et al, 1992).

This synergism is thought to be at least partially due to an increase in intracellular levels of reduced folates, which potentiate the action of 5-fluorodeoxyuridine monophosphate by forming a covalent ternary complex with thymidylate synthase, an essential enzyme for de novo production of the thymidine required for DNA synthesis and repair (Scanlon et al, 1986). Other researchers proposed that 5-FU modulated the repair of platinum-DNA adducts through 5-FU-induced RNA damage, thereby potentiating the anti-tumour activity of CDDP (Esaki et al, 1992). Although these studies directly address the mechanisms by which CDDP and 5-FU synergistically exert anti-tumour activity, it is not known whether these anti-tumour agents can modulate anti-tumour immunity.

*Received 16 April 1998*
*Revised 3 December 1998*
*Accepted 4 December 1998*

*Correspondence to:* K Takizawa

Cancer cells are usually recognized and eliminated by activated T lymphocytes. The adhesive interaction between T lymphocytes and cancer cells is a crucial step for elimination of cancer cells. Several cell adhesion molecules on both T lymphocytes and target cells are believed to be important for anti-tumour immune functions. Intercellular adhesion molecule-1 (ICAM-1), a member of the immunoglobulin superfamily, is a cell adhesion molecule expressed on various types of cancer cells. Its counter-receptor, lymphocyte function associated antigen-1 (LFA-1), a member of the integrin family, is expressed on T lymphocytes (Springer, 1990). The binding of ICAM-1 with LFA-1 is required for a broad range of leucocyte functions, including T-cell-mediated killing of cancer cells, T-helper and B lymphocyte responses, natural killing, antibody-dependent cytotoxicity mediated by monocytes and adherence of leucocytes to endothelial cells and epithelial cells (Martz et al, 1987; Springer et al, 1987). Thus the expression levels of ICAM-1 on cancer cells might be critical for the elimination of cancer cells by T lymphocytes.

In this study we examined whether the anti-tumour drugs CDDP and 5-FU, alone or in combination, can modulate the expression of cell adhesion molecules involved in recognition of cancer cells by T lymphocytes.

### MATERIALS AND METHODS

### Reagents and monoclonal antibodies (mAbs)

CDDP and 5-FU were obtained from Sigma (St Louis, MO, USA). Unconjugated mAbs against the following human antigens were used in this study: anti-ICAM-1 (mouse immunoglobulin (mIg) G2b, LB-2; Becton Dickinson, San Jose, CA, USA), anti-ICAM-2 (mIgG1, B-T1; Immunotech, Marseille, France), anti-ICAM-3 (mIgG2a, HP2/19; Immunotech, Marseille, France), anti-vascular cell adhesion molecule-1 (VCAM-1) (mIgG1, 51-10c9; Pharmingen, San Diego, CA, USA), anti-E-selectin (mIgG, 68-5H11; Pharmingen, San Diego, CA, USA). Staurosporin and genistein were obtained from Wako Pure Chemicals Ltd (Tokyo, Japan). NF-kB SN-50, an inhibitor of nuclear factor kappa B (NF-κB) nuclear translocation, was purchased from Biomol (Plymouth Meeting, PA, USA). Monoclonal mouse anti-human interferon gamma (IFN-γ) antibody were obtained from Genzyme (Cambridge, MA, USA).

### Cell lines and cell culture

NA and HSC-4, cancer cell lines established from patients with SCC of the tongue, were maintained as monolayers in Dulbecco's modified Eagle's medium (DMEM) supplemented with 10% heat inactivated fetal bovine serum (FBS), 100 units $ml^{-1}$ penicillin and 100 μg $ml^{-1}$ streptomycin (complete medium) (Hori et al, 1994). Subconfluent monolayers of NA cells or HSC-4 cells were employed in all experiments.

### Cytotoxic effects of anticancer agents on NA cells

NA cells were seeded in a flat-bottomed 96-well microplate by adding 0.2 ml of complete medium containing $3 \times 10^4$ cells. After 24 h incubation, NA cells were treated with various concentrations of CDDP and/or 5-FU. After a further 18 h incubation, the medium was removed, and cells were washed twice with phosphate-buffered saline (PBS), and fixed and stained for 30 min with 2% ethanol containing 0.2% crystal violet. Bound dye was eluted with 0.1 ml of 1% sodium dodecyl sulphate. Absorbance was measured at 570 nm on a microplate reader MR 5000 (Dynatech) (Kamijo et al, 1989). The percentage of cytotoxicity was calculated from the mean OD by the following equation:

$$\%\ \text{cytotoxicity} = \left(1 - \frac{\text{absorbance (untreated NA cells)} - \text{absorbance (treated NA cells)}}{\text{absorbance (untreated NA cells)}}\right) \times 100$$

### Flow cytometry

Antigens were detected on cell surfaces by indirect immunofluorescence followed by standard flow cytometric analysis. NA cells or HSC-4 cells ($2 \times 10^6$ cells) were incubated in the presence or absence of CDDP and/or 5-FU for 18 h. Cells were washed with PBS at 4°C and incubated with 500 ng to 1 μg of primary antibodies for 30 min at 4°C. Cells were washed three times with PBS followed by a 30 min incubation with the secondary antibody, fluoroscein isothiocyanate (FITC)-conjugated goat antimouse IgG (Becton Dickinson, San Jose, CA, USA). Cells were washed three times with PBS and resuspended with 100–200 μl of 1% paraformaldehyde PBS. Samples were then analysed using a FACscan flow cytometer (Becton Dickinson, San Jose, CA, USA). In some experiments, cells were pretreated with genistein (100 ng $ml^{-1}$), staurosporin ($10^{-9}$ M) or NF-κB SN-50 (18 μM) for 30 min before addition of anticancer agents.

### Reverse transcriptase-polymerase chain reaction

NA cells ($1 \times 10^6$ cells) were incubated with CDDP and/or 5-FU for 4 h. For reverse transcriptase-polymerase chain reaction (RT-PCR) analysis, cytoplasmic RNA was extracted by the guanidine isothiocyanate–phenol–chloroform extraction method. To ensure that the amplification products were from the mRNA being probed, RNA samples were treated with DNase I (Gibco BRL, Gaithersburg, MD, USA) at room temperature for 15 min. First-strand cDNA synthesis was carried out as described previously (Sumitani et al, 1998). Briefly, 1 μg of total RNA was reverse transcribed to single-stranded cDNA in 25-μl of reaction mixture containing 1 × RT buffer (50 mM Tris–HCl pH 8.3, 10 mM potassium chloride, 10 mM magnesium chloride and 10 mM dithiothreitol (DTT)), 0.5 μg of Rnasin (Promega, Madison, WI, USA), 0.5 mM of each dNTP (Pharmacia Biotech, Tokyo, Japan), 2.0 μg of random primer and 5 units of avian myeloblastosis virus (AMV) reverse transcriptase (Gibco BRL, Gaithersburg, MD, USA). The reverse transcriptation reaction mixture was incubated for 90 min at 37°C, heated at 98°C to inactivate the enzyme, and quickly chilled to 10°C. A 5 μl aliquot of cDNA was mixed with 45 μl of PCR mixture containing 1 × PCR buffer, 500 mM of each dNTP, 0.4 mM of each primer and 1 unit of *Taq* DNA polymerase (Promega, Madison, WI, USA). PCR was performed using a programmed temperature control system (Astec, Fukuoka, Japan) set for 35 cycles of denaturation at 94°C for 1 min, annealing at 60°C for 1 min, and extension at 72°C for 2 min. A 10 μl aliquot of each reaction mixture was electrophoresed on a 1% agarose gel, and PCR products were visualized by ethidium bromide staining. To normalize for the amount of input RNA, RT-PCR was performed with primers for the constitutively expressed glyceraldehyde 3-phosphate dehydrogenase (GAPDH) gene.

The primers used for RT-PCR are as follows: ICAM-1: sense, 5′ TATGGCAACGACTCCTTCT 3′; antisense, 5′ CATTCAGCG-TCACCTTGG 3′; amplified product: 238 bp. GAPDH: sense, 5′ TGAAGGTCGGAGTCAACGGATTTGGT 3′; antisense, 5′ CATGTGGGCCATGAGGTCCACCAC 3′; amplified product: 983 bp. IL-1α: sense, 5′ CAAGGAGAGCATGGTGGTAGTAG-CAACCAACG 3′; antisense, 5′ TAGTGCCGTGAGTTTCCCA-GAAGAAGGAGG 3′; amplified product: 491 bp. IL-1β: sense, 5′ ATGGCAGAAGTACCTAAGCTCGC 3′; antisense, 5′ AC-ACAAATTGCATGGTGAAGTCAGTT 3′; amplified product: 801 bp. IFN-γ: sense, 5′ GCATCGTTTTGGGTTCTCTTGGCTG-TTACTGC 3′; antisense, 5′ CTCCTTTTTCGCTTCCCTGTTTT-AGCTGCTGG 3′; amplified product: 427 bp. TNF-α: sense, 5′ GAGTGACAAGCCTGTAGCCCATGTTGTAGCA 3′; antisense, 5′ GCAATGATCCCAAAGTAGACCTGCCCAGACT 3′; amplified product: 444 bp.

### Enzyme-linked immunosorbent assay

NA cells ($4 \times 10^4$ cells in 200 μl of complete medium) were incubated in the presence or absense of CDDP and/or 5-FU for 18 h. Aliquots of culture medium were harvested and cytokine levels in the culture medium were determined using specific enzyme-linked immunosorbent assay (ELISA), (Endogen, Boston, MA, USA), according to the manufacturer's instructions.

### Electrophoretic mobility shift analysis

Electrophoretic mobility shift analysis (EMSA) was performed as previously described (Song et al, 1997). Nuclear extracts were

© 1999 Cancer Research Campaign prepared by treating the cell pellet with cold buffer (10 mmol $l^{-1}$ EGTA, 1 mmol $l^{-1}$ DTT, and 0.5 mmol $l^{-1}$ phenylmethylsulphonyl fluoride (PMSF) for 15 min to allow cells to swell, and then 25 µl 10% NP-40 was added and the nuclei were pelleted. The nuclear pellet was resuspended in buffer (20 mmol $l^{-1}$ Hepes, pH 7.9, 0.4 mol $l^{-1}$ sodium chloride, 1 mmol $l^{-1}$ EDTA, 1 mmol $l^{-1}$ EGTA, 1 mmol $l^{-1}$ DTT and 1 mmol $l^{-1}$ PMSF) for 15 min at 4°C. Debris was pelleted, and supernatants were frozen at –70°C. Nuclear extracts (5–7 µg protein) prepared from NA cells were incubated with 50 000 cpm (0.5 ng) $^{32}$P-end-labelled double-stranded synthetic deoxyoligonucleotide probes for 30 min at room temperature in a 20 ml reaction volume containing 12% glycerol, 12 mmol $l^{-1}$ Hepes–NaOH (pH 7.9), 60 mmol $l^{-1}$ potassium chloride, 5 mmol $l^{-1}$ magnesium chloride, 4 mmol $l^{-1}$ Tris hydrochloride (pH 7.9), 0.6 mmol $l^{-1}$ EDTA (pH 7.9), 0.6 mmol $l^{-1}$ DTT and 1 µg poly (dI)(dC). Protein–DNA complexes were resolved in 5% native polyacrylamide gels pre-electrophoresed for 30 min at room temperature in 0.25 × TBE buffer (22.5 mM Tris–borate and 0.5 mM EDTA, pH 8.3). Gels were dried and exposed overnight to X-ray film (Eastman Kodak, Rochester, NY, USA) with an intensifying screen at –70°C. The oligonucleotide probe used in this study to detect NF-κB was 5′-GCTCCGGAATTTCCAAGC-3′.

### RESULTS

### Cytotoxic effects of CDDP and/or 5-FU on NA cells

Both CDDP and 5-FU dose-dependently exerted cytotoxic activity against NA cells (Figure 1). The concentration of CDDP or 5-FU needed for 50% reduction of the $OD_{570}$ in the control was approximately 500 µg $ml^{-1}$. CDDP and/or 5-FU exert both cytotoxic effect and antiproliferative effect against NA cells. Trypan blue dye exclusion assay revealed that both agents were dominantly cytotoxic at concentrations exceeding 100 µg $ml^{-1}$, though they were antiproliferative and less cytotoxic for NA cells at lower concentrations (data not shown). For subsequent experiments, we employed suboptimal doses of CDDP (100 µg $ml^{-1}$) or 5-FU (100 µg $ml^{-1}$). When both agents (100 µg $ml^{-1}$) were added simultaneously, no synergistic effect was observed (Figure 1). The viabilities of NA cells treated with CDDP (100 µg $ml^{-1}$) and 5-FU (100 µg $ml^{-1}$), alone or in combination, were approximately 99% as determined by trypan blue dye exclusion assays.

### Effects of CDDP and/or 5-FU on the expression of ICAM-1, ICAM-2 and ICAM-3 on NA cells

To determine the effect of CDDP and/or 5-FU on the expression of ICAM-1, ICAM-2 and ICAM-3 on cell surface, we employed flow-cytometric analysis. Cell surface antigen expression on NA cells was evaluated 18 h after incubation with CDDP and/or 5-FU, and the results were compared to untreated control cells. As shown in Figure 2A, ICAM-1 was constitutively expressed on untreated NA cells (mean fluorescence intensity (MFI) was 5.44). Although treatment with CDDP or 5-FU alone for 18 h did not induce the expression of ICAM-1 on NA cells (MFI was 5.71 and 5.97 respectively), expression of ICAM-1 was synergistically induced by treatment with CDDP and 5-FU (MFI was 9.52). Figures 2B and C show that ICAM-2 or ICAM-3 are constitutively expressed on NA cells. In contrast to the expression of ICAM-1, CDDP and/or 5-FU did not up-regulate the levels of ICAM-2 or ICAM-3 on NA cells.

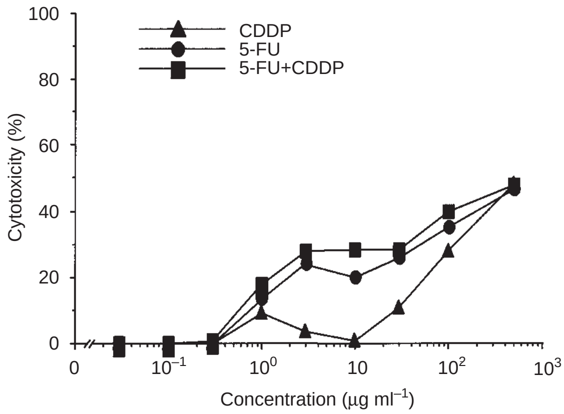

**Figure 1** Cytotoxicity of anticancer agents on NA cells. NA cells were incubated in the presence of various concentrations of CDDP and/or 5-FU for 18 h. Percent cytotoxicity was determined as described in Materials and Methods

### RT-PCR analysis

It was reported that ICAM-1 mRNA can be detected within a few hours after stimulation with IFN-γ in monocytes (Song et al, 1997). Therefore, the expression of ICAM-1 mRNA was examined in NA cells at 4 h after incubation with CDDP and/or 5-FU. To evaluate the effect of CDDP and/or 5-FU on the expression of ICAM-1 mRNA in NA cells, we employed RT-PCR method. RT-PCR analysis revealed that the expression of ICAM-1 mRNA was synergistically induced by CDDP and 5-FU in NA cells, whereas CDDP or 5-FU alone did not induce ICAM-1 mRNA expression (Figure 3A). Densitometrical analysis confirmed the synergistic induction of ICAM-1 mRNA in NA cells treated with CDDP and 5-FU (Figure 3B).

### Effects of protein kinase inhibitors on the expression of ICAM-1 induced by CDDP and/or 5-FU

To determine whether ICAM-1 induction by CDDP and 5-FU is related to protein phosphorylation, NA cells were pretreated with genistein (100 ng $ml^{-1}$) or staurosporin ($10^{-9}$ M) for 30 min. After pretreatment, cells were incubated with CDDP and 5-FU for 18 h, and the expression of ICAM-1 on NA cells were analysed by flow cytometry. Induction of ICAM-1 on NA cells by CDDP and 5-FU was inhibited by the pretreatment with genistein, though pretreatment with staurosporin did not alter ICAM-1 expression on NA cells (Figure 4).

### Effects of CDDP and 5-FU on DNA binding activity on ICAM-1 NF-κB

The ICAM-1 promoter contains NF-κB sites located distal (nucleotides –540 to –528) and proximal (nucleotides –228 to –217) (Song et al, 1997). We employed EMSA to examine whether CDDP and 5-FU induces NF-κB binding activity in NA cells, since recent data indicate that IFN-γ, a potent inducer of ICAM-1 on epithelial cells, increases DNA binding activity of the proximal NF-κB site (Song et al, 1997). Treatment with CDDP and 5-FU increased DNA binding activity of the proximal NF-κB

© 1999 Cancer Research Campaign

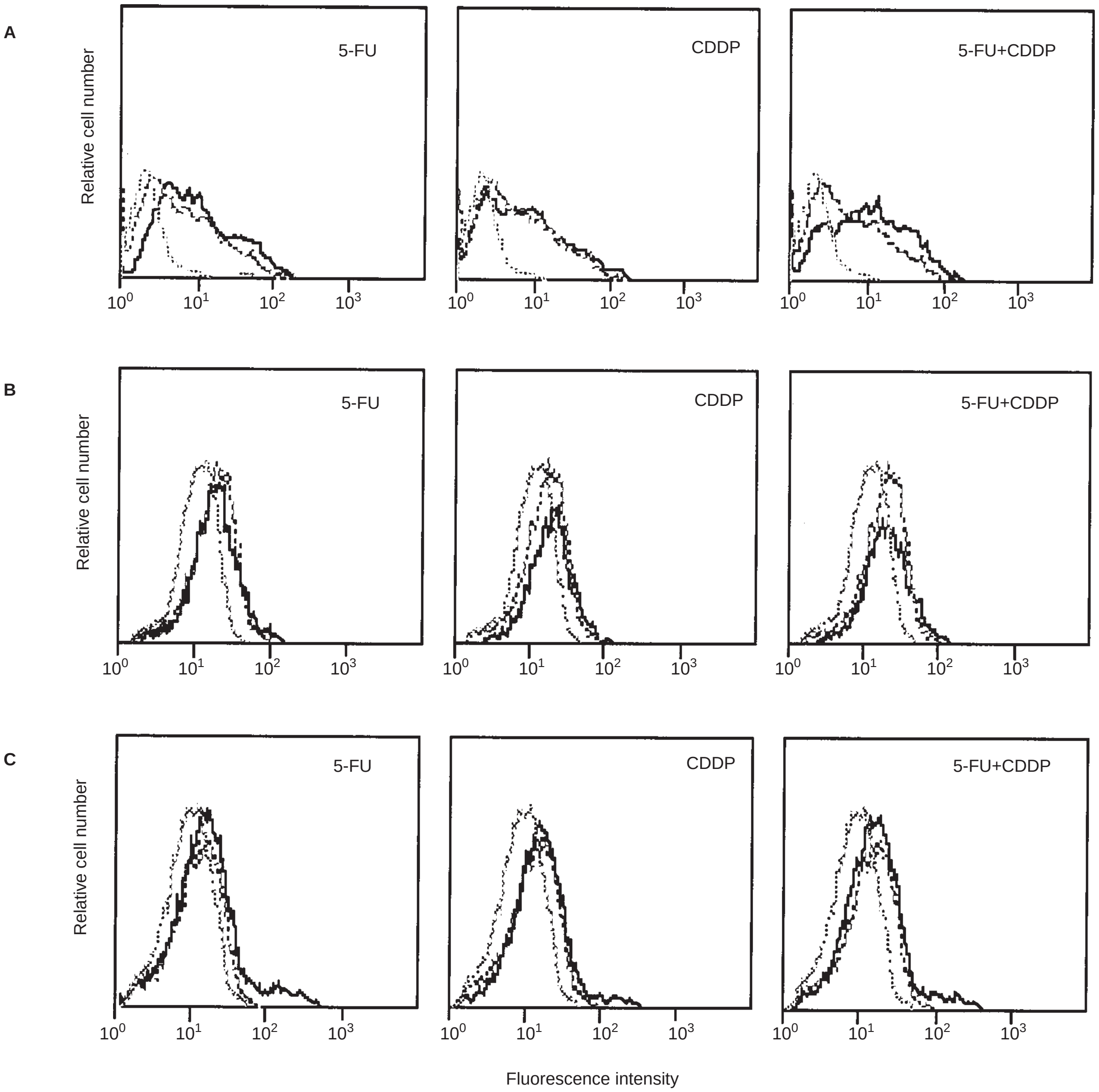

**Figure 2** Effects of anticancer agents on the expression of ICAM-1, ICAM-2 and ICAM-3 on NA cells. NA cells were incubated with CDDP (100 μg ml$^{-1}$) and/or 5-FU (100 μg ml$^{-1}$) for 18 h. The expression of ICAM-1 (**A**), ICAM-2 (**B**) and ICAM-3 (**C**) on NA cells was analysed by flow cytometry as described in Materials and Methods. Negative controls without primary antibody are also shown. (‥) Untreated, (–) Treated, (••) Negative control site (Figure 5), and pretreatment with genistein for 30 min did not inhibit this increase in binding activity (Figure 5). These results demonstrate that CDDP and 5-FU can increase the NF-κB binding activity of the ICAM-1 promoter in NA cells, and this binding activity cannot be inhibited by genistein.

### Effect of the inhibitor of NF-κB nuclear translocation on the CDDP and 5-FU-induced ICAM-1 expression on NA cells

To further investigate the involvement of NF-κB on the induction of ICAM-1 expression by CDDP and 5-FU, NA cells were treated with CDDP and 5-FU in the presence of NF-κB SN-50, a NF-κB nuclear translocation inhibitor, for 18 h. After incubation, the expression levels of ICAM-1 on NA cells were flow cytometrically analysed using a FACscan flow cytometer. Incubation of NA cells with NF-κB SN-50 did not inhibit CDDP and 5-FU-induced ICAM-1 expression (data not shown).

### Effects of CDDP and/or 5-FU on cytokine production by NA cells

ICAM-1 is induced by various stimuli including interleukin-1 (IL-1), tumour necrosis factor-α (TNF-α) and IFN-γ. We examined the effects of CDDP and/or 5-FU on the production of these cytokines by NA cells. NA cells were cultured in the presence or absence of

© 1999 Cancer Research Campaign

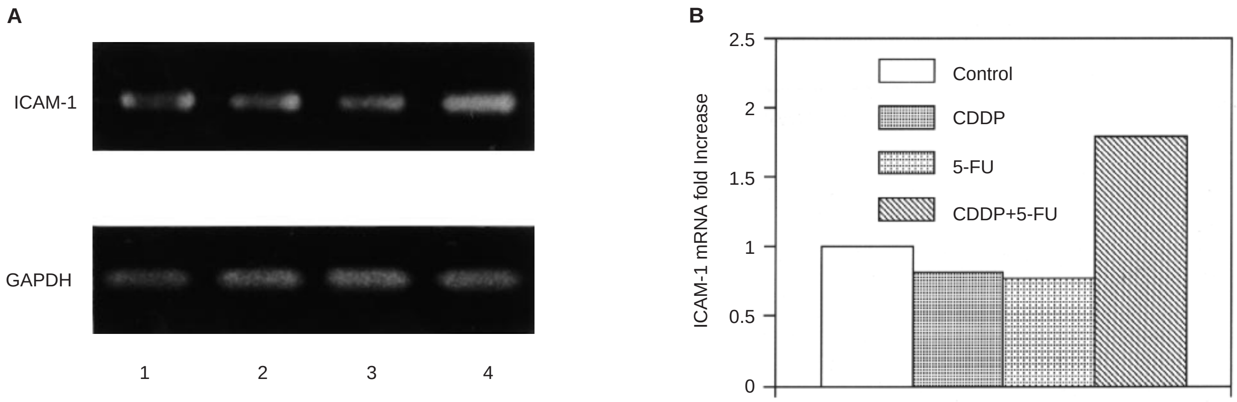

**Figure 3** (**A**) RT-PCR analysis of ICAM-1 mRNA expression in NA cells treated with anticancer agents. NA cells ($1 \times 10^6$) were incubated with CDDP (100 µg $ml^{-1}$) and/or 5-FU (100 µg $ml^{-1}$) for 4 h. Total RNA was extracted, and RT-PCR analysis was performed as described in Materials and Methods. Lane 1, control; lane 2, CDDP; lane 3, 5-FU; lane 4, CDDP and 5 FU. (**B**) Normalized absorption values were obtained by densitometry scanning of ICAM-1 and GAPDH mRNA bands. From the ratio of ICAM-1 to GAPDH, the fold increase over untreated control cells was calculated

CDDP and/or 5-FU for 18 h, and IL-1α, IL-1β, TNF-α and IFN-γ levels in the culture medium were determined by ELISA. All cytokines tested were undetectable in the culture medium of NA cells treated with CDDP and/or 5-FU (data not shown). Accordingly, we employed RT-PCR for analysis of cytokine mRNA levels in NA cells. As shown in Figure 6, IL-1α mRNA and TNF-α mRNA were detectable in untreated NA cells. However, mRNA levels of these cytokines were not up-regulated by treatment with CDDP and/or 5-FU. IL-1β mRNA was undetectable in the total RNA samples extracted from the NA cells treated with CDDP and/or 5-FU (data not shown). Although IFN-γ mRNA level was not induced by CDDP or 5-FU alone, it was synergistically upregulated by simultaneous treatment with CDDP and 5-FU (Figure 6). Then the effect of CDDP and 5-FU on the expression of ICAM-1 on NA cells were examined in the presence of monoclonal antibody against human IFN-γ. NA cells were treated with CDDP and 5-FU for 18 h in the presence of monoclonal antibody against IFN-γ, and the expression of ICAM-1 on NA cells was examined by flow-cytometry. Monoclonal antibody against IFN-γ could not inhibit the induction of ICAM-1 by CDDP and 5-FU on NA cells (data not shown).

### Effects of CDDP and/or 5-FU on the expression of E-selectin and VCAM-1 on NA cells

The expression of E-selectin and VCAM-1 were evaluated 18 h after incubation with CDDP and/or 5-FU, and compared to levels in untreated cells. As shown in Figure 7A, E-selectin was constitutively

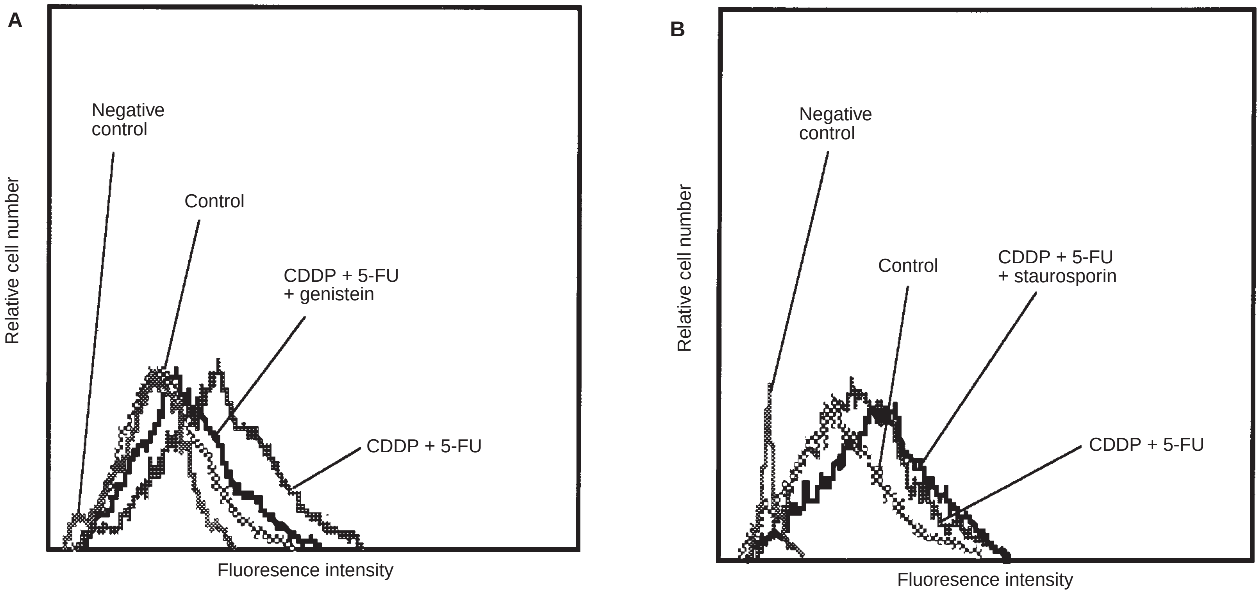

**Figure 4** Effects of protein kinase inhibitors on the expression of ICAM-1 induced by CDDP and 5-FU. NA cells were pretreated with genistein (**A**) or staurosporin (**B**) for 30 min before addition of anticancer agents. After incubaton with CDDP and 5-FU for 18 h, the expression of ICAM-1 on NA cells was analysed by a FACscan flow cytometry as described in Materials and Methods. Negative controls without primary antibody are also shown

 © 1999 Cancer Research Campaign

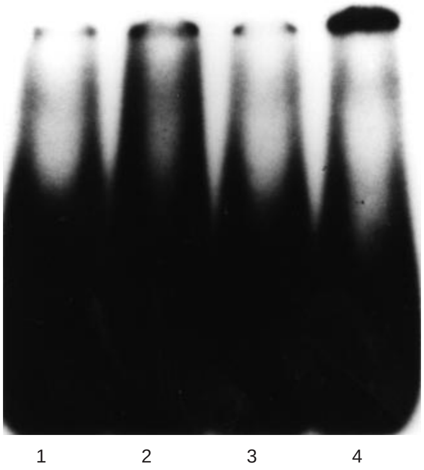

**Figure 5** Electrophoretic mobility shift analysis of nuclear extract prepared from NA cells. After the pretreatment with (lane 3, 4) or without genistein (100 ng ml$^{-1}$, lane 1, 2), NA cells were treated with CDDP (100 μg ml$^{-1}$) and 5-FU (100 μg ml$^{-1}$) for 30 min (lane 2, 4), and nuclear extracts were prepared. Radiolabelled oligonucleotide comprising the proximal ICAM-1 NF-κB site was incubated with 5 mg of nuclear extracts as described in Materials and Methods, and the amounts of specific NF-κB binding complexes were compared to control nuclear extracts

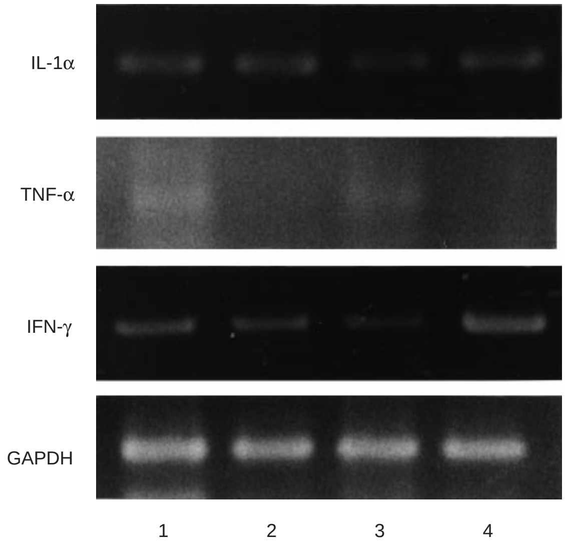

**Figure 6** RT-PCR analysis of cytokine mRNA expression in NA cells treated with anticancer agents. NA cells (1 × 10$^6$) were incubated with CDDP (100 μg ml$^{-1}$) and/or 5-FU (100 μg ml$^{-1}$) for 4 h. Total RNA was extracted, and RT-PCR analysis was performed as described in Materials and Methods. Lane 1, control; Lane 2, CDDP; lane 3, 5-FU; lane 4, CDDP and 5-FU

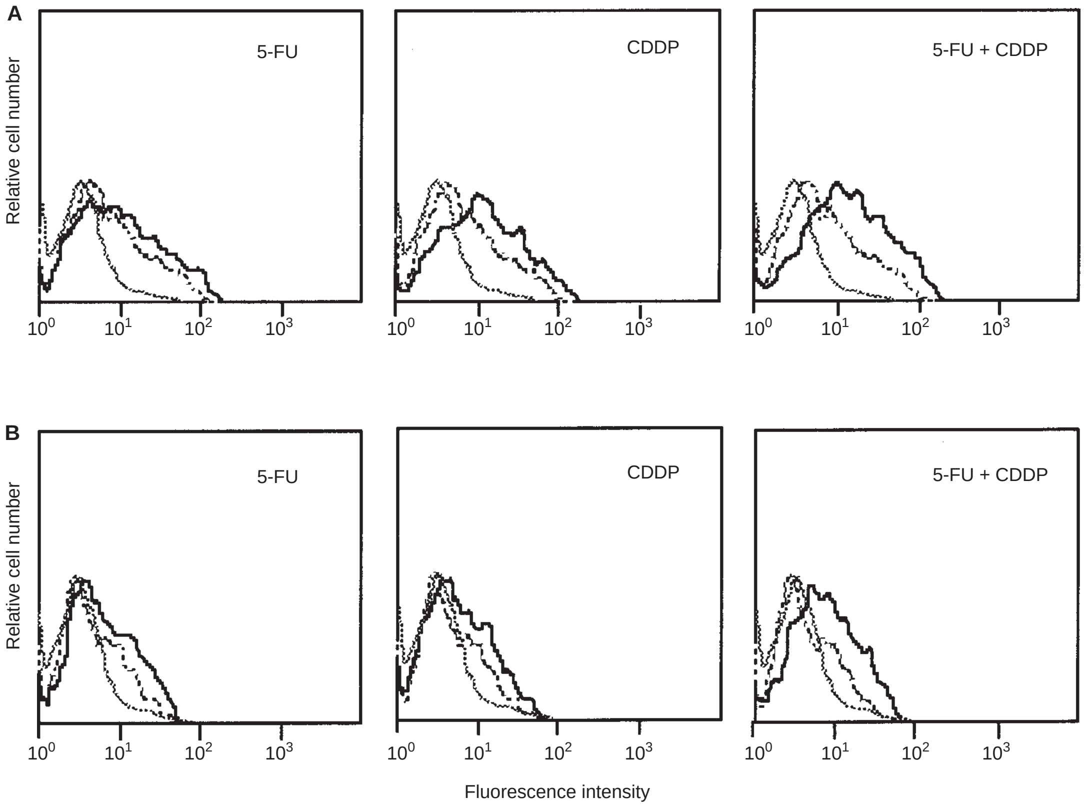

**Figure 7** Effects of anticancer agents on the expression of VCAM-1 and E-selectin on NA cells. NA cells were incubated with CDDP (100 μg ml$^{-1}$) and/or 5-FU (100 μg ml$^{-1}$) for 18 h. The expression of E-selectin (**A**) and VCAM-1 (**B**) on NA cells was analysed by a FACscan flow cytometry as described in Materials and Methods. Negative controls without primary antibody are also shown. (...) Untreated, (–) Treated, (••) Negative control expressed on control cells. Treatment with CDDP or 5-FU slightly induced the expression of E-selectin on NA cells, and expression of E-selectin was additively induced by treatment with CDDP and 5-FU. Similar results were obtained for VCAM-1 expression on NA cells (Figure 7B).

### Effects of CDDP and/or 5-FU on the expression of ICAM-1, ICAM-2 and ICAM-3 on HSC-4 cells

To confirm that the synergistic effects of CDDP and 5-FU reported in this study are not specific to NA cells, the effects of CDDP and/or 5-FU on the expression of ICAM-1, ICAM-2 and ICAM-3 on HSC-4 cells were examined. Since HSC-4 cells were more sensitive for cytotoxicity induced by CDDP and/or 5-FU than NA cells, HSC-4 cells were incubated in the presence of CDDP (10 μg $ml^{-1}$) and 5-FU (100 μg $ml^{-1}$) for 18 h, and cell surface antigen expression on HSC-4 cells was evaluated. As shown in Figure 8A, ICAM-1 was constitutively expressed on untreated HSC-4 cells (MFI was 6.80). Although CDDP or 5-FU alone did not induce the expression of ICAM-1 on HSC-4 cells (MFI was 9.21 and 10.63 respectively), expression of ICAM-1 was synergistically induced by treatment with CDDP and 5-FU (MFI was 20.34). Figures 8B and C show that ICAM-2 or ICAM-3 are constitutively expressed on HSC-4 cells. In contrast to the expression of ICAM-1, CDDP and/or 5-FU did not up-regulate the levels of ICAM-2 or ICAM-3 on HSC-4 cells.

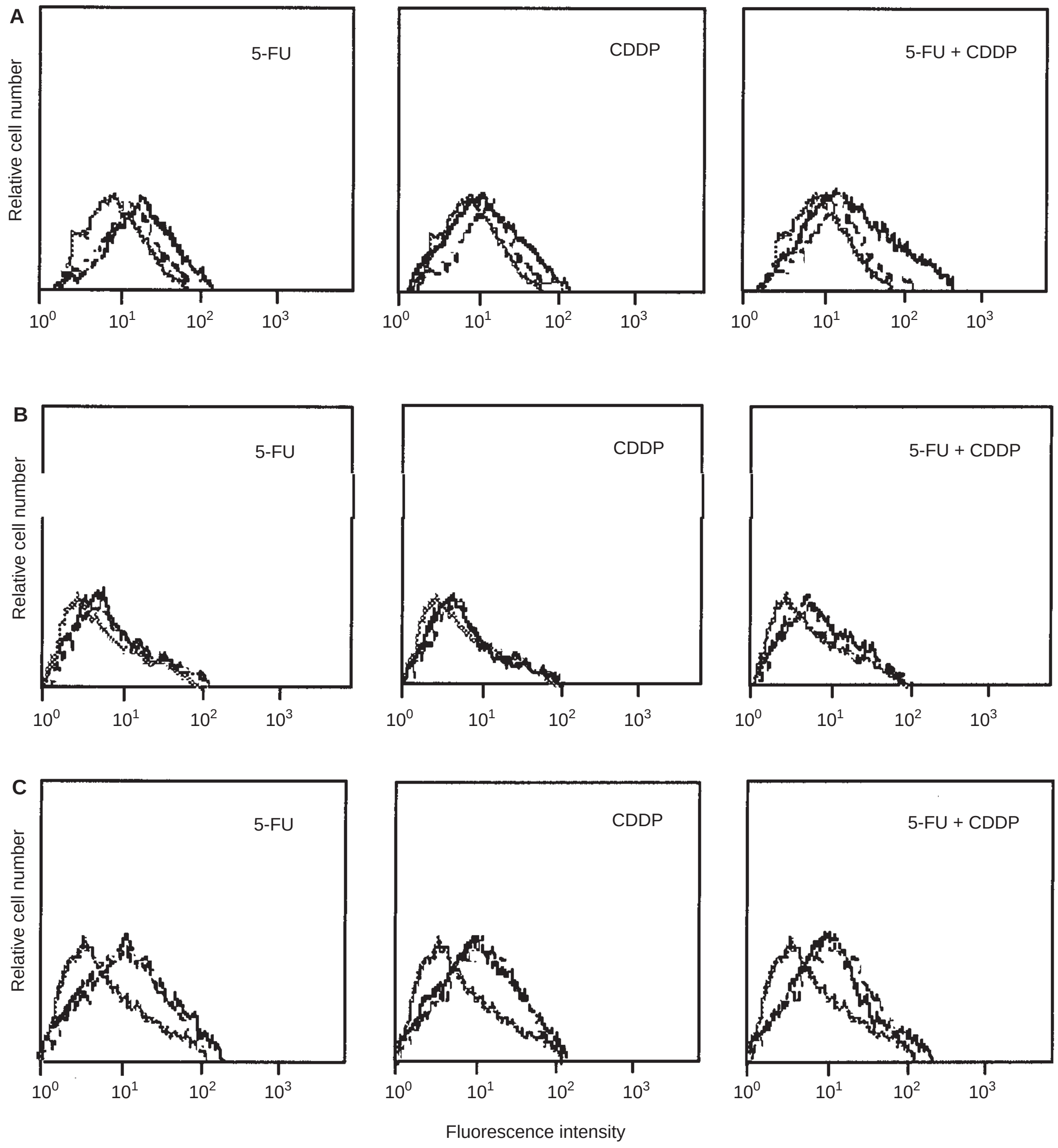

**Figure 8** Effects of anticancer agents on the expression of ICAM-1, ICAM-2 and ICAM-3 on HSC-4 cells. HSC-4 cells were incubated with CDDP (10 μg $ml^{-1}$) and/or 5-FU (100 μg $ml^{-1}$) for 18 h. The expression of ICAM-1 (**A**), ICAM-2 (**B**) and ICAM-3 (**C**) on HSC-4 cells was analysed by flow cytometry as described in Materials and Methods. Negative controls without primary antibody are also shown. (··) Untreated, (–) Treated, (••) Negative control

 *© 1999 Cancer Research Campaign*

### DISCUSSION

Although the principal mechanism of anticancer drugs to eliminate cancer cells is induction of apoptosis (Huschtscha et al, 1996; Ueda et al, 1997), mechanisms to eliminate cancer cells which has escaped from apoptosis, including activation of the host immune system, might also play a role. Previous studies have shown mechanisms by which CDDP and 5-FU synergistically exert anti-tumour activity (Scanlon et al, 1986; Esaki et al, 1992), but it is not known whether these agents can stimulate the host immune system.

Macrophages and T lymphocytes play essential roles in defence against cancer cells. Exclusion of cancer cells in vivo involves several cytotoxic effector mechanisms. For example, secretion of nitric oxide (NO), an unstable free radical gas, is nominated as a major effector molecule for macrophage cytotoxicity. We have recently reported that NO is essential for host immune responses against infectious agents and cancer cells (Kamijo et al 1993*a*, 1993*b*, 1994; Sumitani et al, 1997). Cytolytic T-cells must recognize antigens presented by MHC class I molecules or certain cell adhesion molecules to be effective (Vanky et al, 1990). ICAM-1, an immunoglobulin supergene family member, is found on lymphocytes and monocytes and can be induced on endothelial cells by cytokines, such as IL-1, TNF and IFN (Pohlman et al, 1986; Staunton et al, 1988; Buckle and Hogg, 1990; Springer et al, 1990; Most et al, 1992). Several studies (Vanky et al, 1990; Bouillon et al, 1991; Webb et al, 1991; Ferrini et al, 1994) have indicated that ICAM-1 expression on tumour cells is important for host immune, cell-mediated cytotoxicity. For example, cytokines such as IFN, TNF and IL-1 are able to increase ICAM-1 expression in cancer cell lines in vitro (e.g. HT-29 human colon tumour cells) and are reported to render the cells susceptible to macrophage-mediated killing (Webb et al, 1991). Co-recognition of ICAM-1 and MHC class I is a vital for host cell-mediated tumour cytotoxicity (Vanky et al, 1990).

According to previous reports, CDDP does not alter the expression of cell adhesion molecules on tumour cells (Yamaue et al, 1991; Mizutani et al, 1993). In contrast, it is reported that a combination of CDDP and mitomycin C increases the expression of cell adhesion molecules on Daudi and KATO-III cells (Ishihata et al, 1996). Our results demonstrate that combined treatment with CDDP and 5-FU can elevate the expression of ICAM-1 in a cancer cell line, suggesting that the clinically accepted synergistic anti-tumour effects of CDDP and 5-FU might be, at least partly, mediated by increasing antigen presentation to T-cells.

We also have shown that ICAM-2 and ICAM-3 expression on NA cells is not induced by CDDP and 5-FU. Both ICAM-2 and ICAM-3 bind to LFA-1 on T lymphocytes and activate them, but the activating signals transduced by ICAM-2 and ICAM-3 are weaker than ICAM-1. Thus, induction of ICAM-1 on cancer cells might be more important than induction of ICAM-2 or ICAM-3. In this regard, specific induction of ICAM-1 on cancer cells by CDDP and 5-FU is beneficial in generating optimal antigen presentation of cancer cells to T lymphocytes.

The synergistic effects of CDDP and 5-FU reported in this study are not specific to NA cells, since ICAM-1, not ICAM-2 and ICAM-3, was synergistically induced by CDDP and 5-FU on HSC-4 cells.

In this study, we also investigated the mechanisms by which CDDP and 5-FU induce the expression of ICAM-1 on cancer cells. Treatment of NA cells with genistein, a protein tyrosine kinase (PTK) inhibitor, resulted in abolishment of CDDP and 5-FU-induced ICAM-1 expression, whereas treatment with staurosporin, a protein kinase C inhibitor, did not inhibit ICAM-1 expression induced by CDDP and 5-FU. These results suggest that CDDP and 5-FU induced ICAM-1 expression via a tyrosine related pathway, though further experiments are necessary to isolate the exact mechanism.

We have shown that ICAM-1 regulation by CDDP and 5-FU occurs at the mRNA level. This observation suggests that CDDP and 5-FU target transcription factors interacting with ICAM-1 promoter elements. Previous reports have indicated that ICAM-1 expression is transcriptionally regulated, and the promoter contains binding sites for transcription factors such as NF-κB, AP-1 and C/EBP (Voraberger et al, 1991; Wawryk et al, 1991; Look et al, 1994). Our results indicate that CDDP and 5-FU caused the accumulation of nuclear factor binding to proximal ICAM-1 NF-κB sites. However, suppression of NF-κB nuclear translocation by the inhibitor did not inhibit CDDP and 5-FU-induced ICAM-1 up-regulation. Furthermore, pretreatment with genistein did not reduce the formation of the specific NF-κB binding complex. These results suggest that NF-κB is not important for CDDP and 5-FU synergistic induction of ICAM-1 expression on cancer cells. Future experiments using transfection of the ICAM-1 promoter elements should determine if CDDP and 5-FU-induced ICAM-1 expression is regulated by NF-κB.

An alternative explanation for up-regulation of ICAM-1 may be via an autocrine mechanism involving cytokine. Epithelial cancer cells can produce some cytokines including IL-1α, IL-1β, TNF-α and IFN-γ, and they are reportedly able to induce the expression of ICAM-1 in various types of cells. Thus, NA cells stimulated by CDDP and 5-FU may secret these cytokines, which in turn stimulate NA cells in an autocrine manner to induce the expression of ICAM-1. However, treatment with CDDP and 5-FU did not induce production of either IL-1α, IL-1β or TNF-α in NA cells, as determined by ELISA and RT-PCR analysis. Thus, IL-1α, IL-1β and TNF-α are not involved in up-regulation of ICAM-1. In contrast, IFN-γ mRNA level was up-regulated by simultaneous treatment with CDDP and 5-FU. However, IFN-γ may not be responsible for induction of ICAM-1, since induction of ICAM-1 by CDDP and 5-FU was not inhibited by monoclonal antibody against IFN-γ.

The results of the present study further demonstrate that combined treatment with CDDP and 5-FU directly effects the expression and function of certain molecules engaged in cell–cell recognition and signalling on the surfaces of tumour cells. E-selectin and VCAM-1 are cell adhesion molecules mainly expressed on endothelial cells. They bind to their counter-receptors on cancer cells, and mediate the adhesion of cancer cells to endothelium (Rice and Bevilacqua, 1989; Lauri et al, 1991; Majuri et al, 1992). Previous reports have indicated that the expression of sialyl $Le^x$ and sialyl $Le^a$, counter-receptors for E-selectin, and very late antigen-4 (VLA-4), counter-receptor for VCAM-1, on cancer cells is closely related to their metastatic properties (Majuri et al, 1992; Iwai et al, 1993). Although much attention has been paid to the expression of these cell adhesion molecules on cancer cells, less is known about the expression of E-selectin or VCAM-1 on cancer cells. Interestingly, the expression of E-selectin and VCAM-1 on NA cells was up-regulated by combined treatment with CDDP and 5-FU, though the significance of the induced expression, as well as the functions of these cell adhesion molecules on NA cells, is not clear.

Recently, the existence of soluble forms of ICAM-1 has been demonstrated in the serum of normal subjects, patients with

*© 1999 Cancer Research Campaign* 

autoimmune diseases, transplant rejection and malignancies. The increase in ICAM-1 serum levels in various neoplastic process has been associated with an unfavourable prognosis (Sanchez-Rovita et al, 1998). The effect of CDDP and 5-FU on the level of soluble ICAM-1 in serum of patients with cancer remains to be examined. Increased expression of ICAM-1 on cancer cells by treatment with these anticancer agents suggests they can function as an immune modulator. Their effectiveness as cancer therapeutic agents may be increased if they increase the expression of ICAM-1 on cancer cells without increasing soluble ICAM-1 level.

### ACKNOWLEDGEMENT

This work was supported in part by a Grant-in-Aid for Scientific Research from the Ministry of Education, Science and Culture of Japan.

### REFERENCES

Amrein PC and Weitzman SA (1985) Treatment of squamous cell carcinoma of the head and neck with cisplatin and 5-fluorouracil. *J Clin Oncol* **3**: 1632–1639

Bouillon M, Tessier P, Boulianne R, Destrempe R and Audette M (1991) Regulation by retinoic acid of ICAM-1 expression on human tumor cell lines. *Biochim Biophys Acta* **1097**: 95–102

Buckle AM and Hogg N (1990) Human memory T cells express intercellular adhesion molecule-1 which can be increased by interleukin 2 and interferon-gamma. *Eur J Immunol* **20**: 337–341

Esaki T, Nakano S, Tatsumoto T, Kuroki-Migita M, Mitsugi K, Nakamura M and Niho Y (1992) Inhibition by 5-fluorouracil of cis-diamminedichloroplatinum (II)-induced DNA interstrand crosslink removal in a HST-1 human squamous carcinoma cell line. *Cancer Res* **52**: 6501–6506

Ferrini S, Sforzini S, Cambiaggi A, Poggi A, Meassa R and Canevari S (1994) The LFA-1/ICAM cell adhesion pathway is involved in tumor-cell lysis mediated by bispecific monoclonal-antibody-targeted T lymphocytes. *Int J Cancer* **56**: 846–852

Hori M, Kamijo R, Takeda K and Nagumo M (1994) Downregulation of c-myc expression by tumor necrosis factor-α in combination with transforming growth factor-β or interferon-γ with concomitant inhibition of proliferation in human cell lines. *J IFN Res* **14**: 49–55

Huschtscha LI, Bartier WA, Anderson Ross CE and Tattersall MHN (1996) Characteristics of cancer cell death after exposure to cytotoxic drugs in vitro. *Br J Cancer* **73**: 54–60

Ishihata R, Ejiri Y, Okubo M, Ohara M and Kasukawa R (1996) Enhancement of lymphokine-activated killer cell cytotoxicity implicated in the increased expression of surface adhesion molecules on tumor cells treated with anticancer agents. *Cancer Invest* **14**: 427–434

Iwai K, Ishikura H, Kaji M, Sugiura H, Ishizu A, Takahashi C, Kato H, Tanabe T and Yoshiki T (1993) Importance of E-selectin (ELAM-1) and SIALY LLEWIS[a] in the adhesion of pancreatic carcinoma cells to activated endothlium. *Int J Cancer* **54**: 972–977

Kamijo R, Takeda K, Nagumo M and Konno K (1989) Suppression of TNF-stimulated proliferation of diploid fibroblasts and TNF-induced cytotoxicity against transformed fibroblasts by TGF-β. *Biochem Biophys Res Commun* **158**: 155–162

Kamijo R, Le J, Shapiro D, Havell EA, Huang S, Aguet M, Bosland M and Vilcek J (1993*a*) Mice that lack the interferon γ receptor have profoundly altered responses to infection with bacillus calmette-guerin and subsequent challenge with lipopolysaccharide. *J Exp Med* **178**: 1435–1440

Kamijo R, Shapiro D, Le J, Huang S, Aguet M and Vilcek J (1993*b*) Generation of nitric oxide and induction of major histocompatibility complex class II antigen in macrophages from mice lacking the interferon γ receptor. *Proc Natl Acad Sci USA* **90**: 6626–6630

Kamijo R, Harada H, Matsuyama T, Bosland M, Gerecitano J, Shapiro D, Le J, Koh SI, Kimura T, Green SJ, Mak TW, Taniguchi T and Vilcek J (1994) Requirement for transcription factor IRF-1 in NO synthase induction in macrophages. *Science* **263**: 1612–1615

Kish J, Drelichman A, Jacobs J, Hoschner J, Kinzie J, Loh J, Weaver A and Al-Sarraf M (1982) Clinical trial of cisplatin and 5-FU infusion as initial treatment for advanced squamous cell carcinoma of the head and neck. *Cancer Treat Rep* **66**: 471–474

Kuroki M, Nakano S, Mitsugi K, Lchinose I, Anzai K, Nakamura M, Nagafuchi S and Niho I (1992) In vivo comparative therapeutic study of optimal administration of 5-fluorouracil and cisplatin using a newly established HST-1 human squamous-carcinoma cell line. *Cancer Chemother Pharmacol* **29**: 273–276

Lauri D, Needham L, Martin-Padura I and Dejana E (1991) Tumor cell adhesion to endothelial cells: endothelial leukocyte adhesion molecule-1 as an inducible adhesive receptor specific for colon carcinoma cells. *J Natl Cancer Inst* **83**: 1321–1324

Look DC, Pelletier MR and Holtzman MJ (1994) Selective interaction of a subset of interferon-γ response element-binding proteins with the intercellular adhesion molecule-1 (ICAM-1) gene promoter controls the pattern of expression on epithelial cells. *J Biol Chem* **269**: 8952–8958

Majuri M-L, Mattila P and Renkonen R (1992) Recombinant E-selectin-protein mediates tumor cell adhesion via sialyl-Le[a] and syalyl-Le[x]. *Biochem Biophys Res Commun* **182**: 1376–1382

Martz E (1987) LFA-1 and other accessory molecules functioning in adhesions of T and B lymphocytes. *Hum Immun* **18**: 3–37

Mizutani Y, Bonavida B, Nio Y and Yoshida O (1993) Enhanced susceptibility of cisdiaminedichloroplatinum-treated k562 cells to lysis by peripheral blood lymphocytes and lymphokine activated killer cells. *Cancer* **71**: 1313–1321

Most J, Schwaeble W, Drach J, Sommerauer A and Dierich MP (1992) Regulation of the expression of ICAM-1 on human monocytes and monocytic tumor cell lines. *J Immunol* **148**: 1635–1642

Pohlman TH, Stanness KA, Beatty PG, Ochs HD and Harlan JM (1986) An endothelial cell surface factor(s) induced in vitro by lipopolysaccharide, interleukin 1, and tumor necrosis factor-α increases neutrophil adherence by a Cdw 18-dependent mechanism. *J Immunol* **136**: 4548–4553

Pratesi G, Ginanni L, Manzotti and Zunino F (1988) Sequence dependence of the antitumor and oxic effects of 5-fluorouracil and cis-diamminedichloro-platinum combination on primary colon tumors in mice. *Cancer Chemother Pharmacol* **21**: 237–240

Rice GE and Bevilacqua MP (1989) An inducible endothelial cell surface glycoprotein mediates melanoma adhesion. *Science* **246**: 1303–1306

Sanchez-Rovita P, Jimenez E, Carracedo J, Barneto IC, Ramirez R and Aranda E (1998) Serum levels of intercellular adhesion molecule 1 (ICAM-1) in patient with colorectal cancer: inhibitory effect on cytotoxicity. *Eur J Cancer* **34**: 349–398

Scanlon KJ, Newman EM, Lu Y and Priest DG (1986) Biochemical basis for cisplatin and 5-fluorouracil synergism in human ovarian carcinoma cells. *Proc Natl Acad Sci USA* **83**: 8923–825

Schilsky RL, Choi KE, Vokes EE, Guaspari A, Guarnieri C, Whaling S and Liebner MA (1989) Clinical pharmacology of the stereoisomers of leucovorin during repeated oral dosing. *Cancer* **63**: 1018–1021

Song S, Ling-Hu H, Roebuck KA, Rabbi MF, Donnelly RP and Finnegan A (1997) Interleukin-10 inhibits interferon-g-induced intercellular adhesion molecule-1 gene transcription in human monocytes. *Blood* **89**: 4461–4469

Springer TA (1990) Adhesion receptors of the immune system. *Science* **346**: 425–435

Springer TA, Dustin ML, Kishimoto TK and Marlin SD (1987) The lymphocyte function-associated LFA-1, CD2, and LFA-3 molecules: cell adhesion receptors of the immune system. *Ann Rev Immunol* **5**: 223–252

Staunton DE, Martin SD, Stratowa C, Dustin ML and Springer TA (1988) Primary structure of ICAM-1 demonstrates interaction between members of the immunoglobulin and integrin supergene families. *Cell* **52**: 925–933

Sumitani K, Kamijo R and Nagumo M (1997) Cytotoxic effect of sodium nitroprusside on cancer cells: involvement of apoptosis and suppression of c-myc and c-myb proto-oncogene expression. *Anticancer Res* **17**: 865–872

Ueda M, Kumagai K, Ueki K, Inoki C, Orino I and Ueki M (1997) Growth inhibition and apoptotic cell death in uterine cervical carcinoma cells induced by 5-fluorouracil. *Int J Cancer* **71**: 668–674

Vanky F, Wang P, Patarroyo M and Klein E (1990) Expression of the adhesion molecule ICAM-1 and major histocompatibility complex class I antigens on human tumor cells is required for their interaction with autologous lymphocytes in vitro. *Cancer Immunol Immunother* **31**: 19–27

Voraberger G, Shafer R and Stratowa C (1991) Cloning of the human gene for intercellular adhesion molecule 1 and analysis of its 5′-regulatory region. Induction by cytokines and phorbor ester. *J Immunol* **147**: 2777–2786

 © 1999 Cancer Research Campaign

Wawryk SO, Cockerill PN, Wicks IP and Boyd AW (1991) Isolation and characterization of the promoter region of the human intercellular adhesion molecule-1 *Int Immunol* **3**: 83–93

Webb DS, Mostowski HS and Gerrard TL (1991) Cytokine-induced enhancement of ICAM-1 expression results in increased vulnerability of tumor cells to monocyte-mediated lysis. *J Immunol* **146**: 3682–3686

Yamaue H, Tanimura H, Noguchi K, Iwahashi M, Tsunoda T, Tani M, Tamai, M, Hotta T, Mizobata S and Arii K (1991) Cisplatin treatment renders tumor cells more susceptible to attack by lymphokine-activated killer cells. *J Clin Lab Immunol* **35**: 165–170

*© 1999 Cancer Research Campaign*
